# How Psychiatry Journals Support the Unbiased Translation of Clinical Research. A Cross-Sectional Study of Editorial Policies

**DOI:** 10.1371/journal.pone.0075995

**Published:** 2013-10-16

**Authors:** Hannes Knüppel, Courtney Metz, Joerg J. Meerpohl, Daniel Strech

**Affiliations:** 1 Institute of History and Ethics in Medicine, Centre for Ethics and Law in the Life Sciences – CELLS, Hannover Medical School, Germany; 2 Department of Philosophy, Centre for Ethics and Law in the Life Sciences – CELLS, Leibniz University of Hannover, Germany; 3 German Cochrane Centre, Institute of Medical Biometry and Medical Informatics, University Medical Center Freiburg, Germany; University of Illinois-Chicago, United States of America

## Abstract

**Introduction:**

Reporting guidelines (e.g. CONSORT) have been developed as tools to improve quality and reduce bias in reporting research findings. Trial registration has been recommended for countering selective publication. The International Committee of Medical Journal Editors (ICMJE) encourages the implementation of reporting guidelines and trial registration as uniform requirements (URM). For the last two decades, however, biased reporting and insufficient registration of clinical trials has been identified in several literature reviews and other investigations. No study has so far investigated the extent to which author instructions in psychiatry journals encourage following reporting guidelines and trial registration.

**Method:**

Psychiatry Journals were identified from the 2011 Journal Citation Report. Information given in the author instructions and during the submission procedure of all journals was assessed on whether major reporting guidelines, trial registration and the ICMJE’s URM in general were mentioned and adherence recommended.

**Results:**

We included 123 psychiatry journals (English and German language) in our analysis. A minority recommend or require 1) following the URM (21%), 2) adherence to reporting guidelines such as CONSORT, PRISMA, STROBE (23%, 7%, 4%), or 3) registration of clinical trials (34%). The subsample of the top-10 psychiatry journals (ranked by impact factor) provided much better but still improvable rates. For example, 70% of the top-10 psychiatry journals do not ask for the specific trial registration number.

**Discussion:**

Under the assumption that better reported and better registered clinical research that does not lack substantial information will improve the understanding, credibility, and unbiased translation of clinical research findings, several stakeholders including readers (physicians, patients), authors, reviewers, and editors might benefit from improved author instructions in psychiatry journals. A first step of improvement would consist in requiring adherence to the broadly accepted reporting guidelines and to trial registration.

## Background

The successful translation of findings from clinical trials into health care practice, guidelines and patient information depends on the timely, accurate and unbiased reporting of trial methodology and results. The quality and reporting of clinical trials and systematic reviews can, however, be sub-optimal. Even within the design of RCTs, for example, there is the inherent risk of bias skewing results at various stages and minimizing internal and external validity [1].

First, there is empirical evidence to suggest that lack of, or inadequate attention to, random allocation, allocation concealment, blinding and intention to treat can lead to bias [2], [3]. Second, setting, participants, demographic data, co-medication e.g. can limit the generalizability of the trial results [4], [5]. There is also increasing evidence of selective reporting in clinical trial findings, with some recent examples in pharmacologic treatment for depression and other psychiatric disorders [6], [7], [8], [9].

Since the early 1990s, medical journal editors, methodologists, and clinical researchers have developed reporting guidelines as tools to help improve the quality of reporting in health research articles. A reporting guideline is a checklist, flow diagram, or explicit text to guide authors in reporting a specific type of research, developed using explicit methodology [10]. The first guideline, the CONSORT (CONsolidated Standards Of Reporting Trials) statement, was developed to improve quality of reports on randomized controlled trials; it was first published in 1996, revised in 2001, and updated in 2010 [11], [12]. Reporting guidelines are also available for various other study designs, including diagnostic test accuracy studies (STAndards for Reporting Diagnostic accuracy, STARD) [13], observational studies (STrengthening the Reporting of Observational studies in Epidemiology, STROBE) [14], Meta-analysis Of Observational Studies in Epidemiology (MOOSE) [15], and systematic reviews of randomized controlled trials (Preferred Reporting Items for Systematic reviews and Meta-Analyses, PRISMA) [16].

A recent review of 134 RCTs on pharmacological treatment of bipolar disorder published between 2000 and 2010 found that while some trial-related information is well reported a good part of the reporting quality of RCTs in bipolar disorder falls well below the required level as aimed for by CONSORT [17], [18]. Twenty-five percent (n = 18) of all CONSORT items were generally reported inadequately (reported adequately in less than 25% of all trials). These neglected parts include essential methodological items such as the generation of random allocation sequence (reported in only 24% of all RCTs), method of allocation concealment (in 22%), and all items relevant to the randomization implementation. Also, information with essential clinical relevance was generally reported inadequately, such as the effect size (in 22%) and the number needed to treat (16%). Other analyses of the quality of reporting in psychiatry journals have made similar findings [19], [20], [21].

The poor quality of reporting combined with the selective reporting of trial findings undermines timely, accurate and unbiased translation of trial results in health care practice. It has been shown, firstly, that entire trials with primarily negative results were not published at all (publication bias) [22]. Secondly, it has more recently been shown that some published trials report information selectively, with the effect of prioritizing the benefit of a medical measure or suppressing the results concerning its potential harm[23], [24]. There is a consensus in medical research, in publication ethics and among the leading scientific journals that trial registration currently represents the best strategy for countering selective publication or making it suitably transparent [24], [25], [26]. Trial registers have existed since the 1960s [27]. The most significant registries at present are ClinicalTrials.gov, run by the National Library of Medicine (USA), which has been accepting clinical trials outside the USA since 2005, and the registry network of the WHO, the International Clinical Trials Registry Platform (ICTRP), which has been in operation since 2007.

The *Uniform Requirements for Manuscripts Submitted to Biomedical Journals* (URM) developed by the *International Committee of Medical Journal Editors* (ICJME) [28] require, first, that authors consult reporting guidelines relevant to their specific research design (such as CONSORT for RCTs, or other tools that can be identified at the website of the EQUATOR network (www.equator-network.org) and, second, that trials are registered in a public trials registry.

While the responsibility for improvement of unbiased reporting should primarily lie with the investigators, reviewers and journal editors could facilitate the process by encouraging authors to consider reporting guidelines and to register their trials. Whether reporting guidelines are being endorsed and implemented by medical journals has been studied for general medicine [29], [30], pediatrics and urology [31], [32], [33], [34].

Although inadequate quality of reporting and selective reporting of trial data have often and recently been demonstrated for psychiatric disorders [6], [7], [8], [9], [17], [19], [20], [21], no study has so far investigated the extent to which author instructions in psychiatry journals endorse reporting guidelines and trial registration as encouraged by the URM.

This study aimed to analyze whether author instructions and instructions during the submission procedure of psychiatry journals mention, recommend, or require 1) the adherence to the URM as published by the ICMJE; 2) the use of major reporting guidelines; and 3) trial registration.

## Methods

Based on Journal Citation Reports from 2011 we identified 130 journals indexed in the subject category “psychiatry”. We also identified a subsample of 10 psychiatry journals with the highest impact factor (“top-10”). We restricted our analysis to Journals published in English or German. We accessed the “author’s instructions” or similar texts on the journals’ websites between July and August 2012. We further accessed the instructions given during the online submission procedure in September 2012. The online submission procedures were entered by a fake submission of an “original paper” or a “clinical research”, “clinical trial” paper. All PDFs or website texts were downloaded using WinHTTrack 3.46-1 for documentation.

Two authors independently assessed whether the author instructions mention the URM, major reporting guidelines (CONSORT, STARD, STROBE, MOOSE, and PRISMA) and trial registration. The QUOROM (QUality Of Reporting Of Meta-analyses) guideline was updated and renamed PRISMA in 2009; for this analysis, we classified QUOROM as a subgroup of PRISMA. The rating options were 1) “not mentioned”, 2) “mentioned” (without specification) 3) “consideration recommended” or 4) “consideration required”. The rating “consideration recommended” was applied to moderate wording in the author instructions such as “should” or “we recommend that…”. The rating “consideration required” was applied to strong wording like “authors must …”, “we expect authors to …” or “we require authors to…”.

If two or more journals referred to the same author instructions (e.g. because of the same publisher), they were treated as independent journals for evaluation.

We accessed the ICMJE website in September 2012 to identify which journals are listed as following the URMs.

We calculated frequency data by standard descriptive statistics.

## Results

After exclusion of 7 psychiatry journals due to language restriction or the lack of any web page we included 123 journals in our analysis (116 in English and 7 in German language).

### Author’s instructions regarding URM and ICMJE policies

From the 123 psychiatry journals 21% (n = 26) recommend or require following the URM and another 34% (n = 42) “only” mention the URM at some point in their author instructions or during the online submission process (see table 1).

**Table 1:** Author instructions regarding the Uniform Requirements for Manuscripts (URM) developed by the ICMJE.

ICMJE (URM)	Not mentioned	Mentioned without specification	Mentioned with recommendation to adhere	Mentioned with requirement to adhere
Psychiatry Journals (n = 123)	55 (45%)	42 (34%)	9 (7%)	17 (14%)
Top-10 Psychiatry Journals (n = 10)	1 (10%)	-	1 (10%)	8 (80%)

In contrast, 90% of the top-10 psychiatry journals recommend or require adherence to ICMJE’s URMs.

Of the 123 psychiatry journals, 11 are listed on the ICMJE website among other journals that have requested inclusion on the list of publications that follow the ICMJE’s URMs. However, 2 of these 11 journals mention neither reporting guidelines nor trial registration in their author instructions or during their online submission process.

### Author’s instructions regarding reporting guidelines

The CONSORT statement, which guides the reporting of randomized controlled trials (RCTs), was most prominently mentioned in the journals’ author instructions (see table 2).

**Table 2:** Authors instructions regarding reporting guidelines.

Reporting Guidelines	Psychiatry Journal (n = 123)				Top-10 Psychiatry Journals (n = 10)
	Not mentioned	Mentioned without specification	Mentioned with recommendation to adhere	Mentioned with requirement to adhere	Mentioned with recommendation OR requirement to adhere
**CONSORT** (RCTs)	89 (72%)	6 (5%)	20 (16%)	8 (7%)	5 (50%)
**PRISMA/ QUOROM** (Systematic Reviews/Meta-analyses of RCTs)	114 (93%)	1 (1%)	6 (5%)	2 (2%)	1 (10%)
**STROBE** (Observational studies)	117 (95%)	1 (1%)	2 (2%)	3 (2%)	-
**MOOSE** (Systematic Reviews/ Meta-analyses of observational studies)	117 (95%)	3 (2%)	1 (1%)	2 (2%)	1 (10%)
**STARD** (Diagnostic accuracy studies)	117 (95%)	2 (2%)	3 (2%)	1 (1%)	2 (20%)

For all psychiatry journals 23% (n = 28) and for the top-10 psychiatry journals 50% either recommended or required adherence to CONSORT. All other reporting guidelines were recommended or required in 3% to 7% of all psychiatry journals and in 0% to 20% of the top-10 psychiatry journals (see table 2).

### Author’s instructions regarding trial registration

Of all 123 psychiatry journals, 34% (n = 42) and for the top-10 psychiatry journals 70% explicitly recommend or require the authors to register clinical trials. Only 13 of these (11% of all 123 journals and 30% of the top-10 journals) require the registration number during their online submission process (see table 3).

**Table 3:** Author instructions regarding trial registration.

Trial registration	Not mentioned (not even indirect via mentioning ICMJE)	Mentioned with recommendation to adhere	Mentioned with requirement to adhere
Psychiatry Journals (n = 123)	81 (57)/66% (46%)	11 (9%)	31 (25%)
Top-10 Psychiatry Journals (n = 10)	3 (30%)	-	7 (70%)

Furthermore, only 12% (n = 15) of all psychiatry journals and 60% of the top-10 journals mention specific trial registries. In total, eleven different trial registries were mentioned with clinicaltrials.gov as the most prominent (n = 14).

### Comparison among clinical specialities

The results for all psychiatry journals are similar to overview findings in other specialities like paediatrics and urology that applied assessment tools similar to those applied in this study [31], [32], [33], [34]. One author of this study (JM) also contributed to the editorial policy analyses in paediatrics and urology (see table 4).

**Table 4:** Comparison of findings among clinical specialties.

Policies	Clinical specialities (with percentages of journals that mentioned without specification, recommend or require adherence to the respecting policies)		
	Psychiatry (n = 123)	Urology (n = 55) [31], [34]	Pediatrics (n = 69) [32]
ICMJE (URM)	54%	58%	55%
CONSORT	23%	24%	20%
MOOSE	3%	6%	4%
PRISMA/QUOROM	7%	5%	6%
STARD	3%	6%	6%
STROBE	4%	5%	4%
Trial Registration	34%	36%	23%

## Discussion

Several internationally agreed policies and tools aim to improve the unbiased translation of research findings into clinical practice and health policy decision-making. Core policies and tools in this respect are 1) the URMs (uniform requirements for manuscripts submitted to biomedical journals) drafted by the ICMJE, 2) reporting guidelines (e.g. CONSORT, STROBE, PRISMA) collated by the EQUATOR network, and 3) trial registries such as clinicaltrials.gov run by the United States National Library of Medicine (NLM) at the National Institutes of Health or registries certified by the WHO and working with the International Clinical Trials Registry Platform (ICTRP).

Our main finding is that only a minority of all psychiatry journals (n = 123) recommend or require 1) following the URM (21%), 2) adherence to reporting guidelines such as CONSORT, PRISMA, STROBE (23%, 7%, 4%), or 3) registration of clinical trials (34%). While the top-10 psychiatry journals (ranked by impact factor) highlight core recommendations and requirements more frequently (URM = 90%, CONSORT = 50%, trial registration = 70%) there is still room for improvement. Beside the fact that three top-10 journals do not recommend or require trial registration only one top-10 journal recommends or requires authors to follow the PRISMA statement that aims to support reporting of systematic reviews of clinical trials.

It is obvious that authors are accountable for their manuscripts, and it is their obligation to prepare their research articles in an accurate, transparent, and complete manner so that all the information important for data interpretation is available. However, we suspect that many authors will neither know the recommendations given in the ICMJE’s URMs, nor reporting guidelines such as CONSORT or the practical and ethical rationale for registering clinical trials.

One first reason for scientific journals to include information in their author instructions about reporting guidelines and trial registries is to help potential authors to refine the scientific strength and impact of their publications. Authors are not only interested in the publication of papers. Academic remits more and more refer to how the scientific community judges the content of papers. For example, post-publication reviews and the number of citations are becoming more important for academic careers and grant proposals. Nevertheless, beside the intrinsic motivation of researchers the unbiased translation of research findings also depends on its consistent and rigor promotion. Thus, strong wording in editorial policies that require trial registration and the application of reporting guidelines is necessary but not sufficient. The adherence to such requirements should be made verifiable, for example by requiring the inclusion of the trial registration number in the manuscript. At present, however, 70% of the high impact journals in Psychiatry do not ask for the specific trial registration number.

Furthermore, it is questionable whether the peer-review process is sufficient to guarantee completeness and accuracy of funded research [35] and good reporting quality [31]. Because better structured papers that do not lack substantial information can improve readability, reviewers and readers might also benefit from author instructions that help to improve reporting quality.

As well as authors, journals might also have an interest in adhering to internationally agreed and broadly accepted quality standards. We currently face controversial discussions about the best way to organize scientific publication. Public institutions discuss whether to sponsor open access publications. Against this background, journals that do not support and promote basic measures to improve the readability and credibility of publications may struggle to remain viable in the near future. Public financing of open access publications should require that journals which classify for reimbursement of publication fees include information about reporting guidelines and trial registration in their author instructions and during their online submission process.

Independently of the personal interests of researchers and journal editors, good science should primarily aim to decrease biased publications of information that can negatively influence clinical and public health decision-making. For example, the validity of systematic reviews and meta-analyses that synthesize findings from original studies will be undermined by biased or poorly reported research findings. Finally, the core principles of medicine (including the Ethics Codex of the APA) such as non-maleficence, respect of autonomy and justice all demand greater efforts by journal editors to improve the quality of reporting and trial registration [36].

For the field of psychiatry, which addresses an immense patient population with one of the world’s highest burdens of disease, major improvements have to be made with respect to how the majority of journals inform and require their authors to adhere to a high quality of reporting and adequate trial registration. Our review indicates that the top-10, high impact psychiatry journals demonstrate more interest in high quality publications. But also among these flagships of psychiatry journals more could be done to enforce the registration, improve the reporting, and finally facilitate unbiased translation of clinical research findings.
